# The Refusal of Wisconsin

**Published:** 1899-10

**Authors:** 


### THE REFUSAL OF WISCONSIN.

There was a general feeling of satisfaction felt, at the recent meeting of the National Association of Dental Faculties, that at last a harmonious settlement had been reached with the National Association of Dental Examiners, and this feeling at Niagara has been reflected upon the profession, and, as far as the limited time since that meeting has permitted, there has been a general response of satisfaction with the result.

It was, therefore, with deep regret that the Wisconsin Board of Dental Examiners has seen fit to refuse to be bound by this settlement, and has determined upon a course that must, if persisted in, intensify the spirit of antagonism that was supposed to be happily settled for all future time.

It was this hope that influenced the majority of the members of the Association of Faculties to abstain from any legislation that would seem, in the least degree, to lessen the harmony existing between the two national bodies.

The fear existing in the minds of the few that there might be State Boards that would refuse to endorse the action of the National Association of Dental Examiners led to an effort to have a standing Committee on Law, prepared to meet all such contingencies. The wisdom of this is now made evident by the repudiation of Wisconsin of this settlement and an expressed determination not to withdraw litigation, but to permit the matter to be settled in the higher court. All this is exceedingly discouraging, for if the example set by this State is followed by others, it means a renewal of the legal warfare that can only end in injury to all concerned.

From recent information there is a slight hope that Wisconsin will review its decision and honorably retrace its steps and fulfil the promise made at Niagara in the presence of the two contracting parties.

If this be not done, there can be but one course for the National Association of Dental Faculties to pursue, and that is, to resist the encroachments of this board, and others that propose to follow a similar course, to the last legal extremity.

The colleges of this country have a duty to perform to those who have received their diplomas, a duty that cannot be laid aside. The graduated students have fulfilled their portion of the contract, and it remains for the colleges to do theirs.

It was thought at the Niagara conference that the National Association of Dental Examiners possessed a controlling power over the State Boards represented, and while these were comparatively few compared with the States possessing laws governing the practice of dentistry, it was nevertheless thought that, even with those outside of its direct influence, there would be a moral effect which would result in harmonizing all differences. The first response to that agreement came from Wisconsin, and seems to dispel this illusion. Within a month after the adjournment of these two contracting bodies this State repudiates its authority, and another—New Jersey—has resigned membership. Thus the dental colleges are practically where they were prior to the Niagara Falls conventions.

While it would be pleasant to feel that the National Association of Examiners represented the various State Boards of the country, the facts warrant the assumption that it represents but a small portion of the States, and that it seems incapable of controlling these so absolutely that the Faculties Association can rely upon it for protection. In stating this it is recognized that the worthy men who assembled at Niagara are not to blame for this state of affairs. They acted the noble part, but it must be evident to them, as it is to others, that power did not exist in the Association of Examiners to fulfil their part of the contract.

It is with no wish to disturb the amicable relations supposed to have been established at the recent meeting, but it becomes a duty to warn State Boards that as the olive branch was honestly offered and accepted, and if the covenant which it represents be broken, as it is feared it may be, there can be but one result, and that continued litigation. *The colleges must protect their diplomas.* They cannot consent to permit outside parties, however panoplied with political power, to dictate to them in the manner attempted. The effort must be resisted, if it requires the combined power of the colleges to accomplish it. While this is true, it is hoped that wise counsels will prevail, and that the colleges may not be obliged to accept the gage of legal battle, but better that than a tame servility to a class of men whose interest in dental education may equal the police powers they possess to annoy and hinder its progress. If Wisconsin can find a way out of this difficulty, peace may rest within our borders; but if not, the faculties of the various colleges may rest assured that lasting peace can only be secured by a determined war upon all obstructionists, and that this can only be accomplished by a united and determined effort.

